# Physiological Associations, Within-Session Repeatability, and Exploratory Prediction of Peak Oxygen Uptake Using the Revised 3-Minute Seated Stepping Test in Healthy Young Japanese Adults

**DOI:** 10.3390/jfmk11040388

**Published:** 2026-09-27

**Authors:** Takashi Oshima, Kenichiro Tanaka, Shinya Nakagawa, Ami Tominaga, Miku Matsuo, Masaharu Asai

**Affiliations:** 1 Physical Therapy Research Field, Graduate School of Medicine, Oita University, Yufu 879-5593, Oita, Japan; m23d9007@oita-u.ac.jp; 2 Department of Physical Therapy, Oita Rehabilitation College, Oita 870-8658, Oita, Japan; 3 Physical Therapy Course, Faculty of Welfare and Health Sciences, Oita University, Oita 870-1192, Oita, Japan; tanaka-kenichiro@oita-u.ac.jp; 4 Department of Rehabilitation, Oita Nakamura Hospital, Oita 870-0044, Oita, Japan; 5 Department of Rehabilitation, Soejima Orthopedic Clinic, Takeo 843-0024, Saga, Japan; caixinfuyong@gmail.com; 6 Department of Rehabilitation, Yohkoh Rehabilitation Hospital, Sasebo 857-0022, Nagasaki, Japan; green.m.35@icloud.com

**Keywords:** cardiorespiratory fitness, peak oxygen uptake, cardiopulmonary exercise testing, seated stepping test, submaximal test

## Abstract

**Background and Objectives**: Cardiopulmonary exercise testing (CPET) is a reference standard for cardiorespiratory fitness but requires specialized equipment and trained personnel. The Revised 3-Minute Seated Stepping Test (r3MSST) requires minimal space and equipment. This study examined its physiological associations, within-session repeatability, and exploratory prediction of peak $\dot{V}$O_2_ in healthy young adults. **Methods**: Fifty-seven healthy young adults completed the r3MSST and maximal CPET. Associations with CPET-derived variables were examined using Spearman’s rank correlations. A peak $\dot{V}$O_2_ prediction model was developed using multiple regression and internally validated by bootstrap resampling. Repeatability was assessed using the intraclass correlation coefficient (ICC), standard error of measurement (SEM), minimal detectable change at the 95% confidence level (MDC_95_), and Bland–Altman analysis. **Results**: Peak $\dot{V}$O_2_ during the r3MSST was strongly associated with CPET-derived peak $\dot{V}$O_2_ (r_s_ = 0.725, 95% CI: 0.567–0.831, *p* < 0.001). The model explained 85.8% of peak $\dot{V}$O_2_ variance (R^2^ = 0.858); bootstrap validation yielded optimism-corrected R^2^ = 0.788, root mean square error (RMSE) = 269.4 mL/min, and calibration slope = 0.960. Repeatability was uncertain (ICC = 0.774, 95% CI: 0.231–0.911; SEM = 79.1 steps; MDC_95_ = 219.2 steps); Bland–Altman analysis showed a mean difference of −82.8 steps (95% limits of agreement, −250.5 to 84.9). **Conclusions**: This study provides preliminary evidence that physiological responses during the r3MSST are associated with CPET-derived measures of exercise capacity in healthy young adults; however, the association between step count and CPET-derived peak $\dot{V}$O_2_ was weak, its incremental predictive contribution was limited, and repeatability showed a systematic difference between trials. Further studies should establish between-day reliability, external predictive validity, and applicability in clinical populations.

## 1. Introduction

Cardiorespiratory fitness (CRF) is a key indicator of functional capacity and overall health and is strongly associated with morbidity and mortality [1]. Cardiopulmonary exercise testing (CPET) is considered the reference standard for evaluating CRF because it provides an integrated assessment of the pulmonary, cardiovascular, and skeletal muscle responses during exercise [2]. However, CPET is not always feasible in routine clinical practice. Among the physiological variables obtained during CPET, peak oxygen uptake (peak $\dot{V}$O_2_) is widely accepted as the most robust measure of cardiorespiratory fitness and has important diagnostic, prognostic, and therapeutic implications [2]. Consequently, peak $\dot{V}$O_2_ is widely used to evaluate exercise capacity, guide exercise prescription, and assess treatment response [2,3].

Despite its clinical value, CPET has several practical limitations. The test requires specialized equipment, trained personnel, and considerable time for administration and interpretation, making it difficult to implement routinely in many hospitals, outpatient rehabilitation facilities, and community-based healthcare settings [2,4]. Consequently, field exercise tests have become increasingly important when CPET is unavailable [1,4]. Field exercise tests are widely used to evaluate exercise capacity and monitor rehabilitation outcomes [3,4]. Recent studies have further supported the use of field exercise tests for assessing exercise capacity. Walking and stepping tests have been developed to estimate maximal oxygen uptake in healthy adults [5], while a recent systematic review has highlighted the use of various field exercise tests, including step tests, for evaluating exercise capacity in clinical populations [6]. The six-minute walk test (6MWT) and the incremental shuttle walk test (ISWT) are among the most widely used field exercise tests because they are valid, reliable, and responsive measures of functional exercise capacity [4,7]. A reference equation for the 6MWT has been reported for Japanese adults aged 40–79 years, and a reference equation for the ISWT has been established for healthy Japanese adults, facilitating the interpretation of exercise capacity in clinical practice [8,9]. Although the 6MWT and ISWT are well-established field exercise tests, both require adequate walking space and sufficient walking ability, which may limit their applicability in individuals with impaired mobility or in space-restricted clinical settings [4]. Therefore, there remains a need for field exercise tests requiring minimal space and providing meaningful estimates of exercise capacity. A seated stepping test has the potential to address these practical limitations while maintaining the advantages of a simple field-based assessment.

The Revised 3-Minute Seated Stepping Test (r3MSST) was developed by modifying the original 3-Minute Seated Stepping Test (3MSST) to improve test standardization and practical applicability. Previous studies suggested that the original 3MSST may be useful for evaluating exercise capacity in both patients undergoing cardiac rehabilitation and healthy adults [10,11]. However, evidence regarding its ability to estimate peak $\dot{V}$O_2_ remains limited because only a small preliminary study in healthy young men has been conducted [11]. Furthermore, the relationship between r3MSST performance and CPET-derived peak $\dot{V}$O_2_ and the within-session repeatability of the r3MSST have not yet been established. Accordingly, further investigation is warranted to determine its utility as a practical field exercise test for assessing exercise capacity.

Therefore, this study aimed to examine the physiological associations and within-session repeatability of the r3MSST and to explore the prediction of peak $\dot{V}$O_2_ in healthy young Japanese adults. Specifically, we examined its association with CPET-derived peak $\dot{V}$O_2_, evaluated its within-session repeatability, and developed and internally validated a prediction equation for estimating peak $\dot{V}$O_2_ using r3MSST step count and selected participant variables. Establishing these measurement properties in healthy young adults represents an essential first step toward future evaluation of the r3MSST in broader populations.

## 2. Materials and Methods

### 2.1. Participants

The required sample size was calculated using G*Power version 3.1.9.7 based on a multiple regression analysis assuming a medium effect size (f^2^ = 0.15), five predictors, a 5% significance level (α = 0.05), and 80% power; the required sample size was estimated at 55 participants. The five predictors used for this power calculation represented the assumed number of predictors for sample-size estimation and did not correspond to five specific variables prespecified for the final prediction model. This power calculation was not intended to establish the sample size required for prediction-model development following data-driven variable selection or for precise estimation of reliability.

Students at Oita University and Oita Rehabilitation College were invited to participate between November 2024 and October 2025; the total number of students approached was not recorded. Fifty-seven individuals underwent eligibility assessment, all of whom met the eligibility criteria and were enrolled in the study. No participants were excluded or withdrew after enrollment, and there were no missing observations; therefore, all 57 participants were included in the final analyses. The participants were healthy young adults (aged 18–23 years).

### 2.2. Eligibility Criteria

No specific positive inclusion criteria, including an age criterion, were predefined; students who volunteered to participate were assessed according to the exclusion criteria described below. The observed age range of 18–23 years reflected the characteristics of the recruited student population rather than a predefined age-based eligibility criterion. Participants were excluded if they (1) had cardiovascular conditions contraindicating CPET, (2) had respiratory diseases, (3) were unable to exercise safely because of musculoskeletal disorders, or (4) did not provide informed consent.

### 2.3. Ethical Considerations

This study was conducted in accordance with the Declaration of Helsinki. All participants were provided with a participant information sheet describing the purpose and procedures of the study, and each participant provided written informed consent by signing the consent form before the measurements were performed. The consent form was prepared in duplicate, with one signed copy retained by the participant and the other by the principal investigator. The study protocol was approved by the institutional ethics committee of the Faculty of Welfare and Health Sciences, Oita University (Approval No. F240028; date of approval: 23 October 2024).

### 2.4. Measurements

Data were collected from February 2025 to February 2026. Measurements of participants recruited by October 2025 were subsequently conducted according to participants’ availability, with data collection continuing until February 2026. Baseline characteristics, including age, height, and body mass, were recorded. Medical history was obtained through an interview to identify underlying conditions relevant to the exclusion criteria.

Lower-extremity muscle strength was assessed using a dynamometer (μTas F-2, ANIMA Co., Tokyo, Japan). Measurements were obtained with participants seated and the hip and knee joints flexed at 90°. Knee extensor strength was measured with the sensor pad positioned on the anterior distal lower leg, whereas hip flexor strength was measured with the pad positioned on the anterior distal thigh. Maximal voluntary isometric contractions were performed twice per muscle group, and the highest value was recorded. All measurements were conducted by the same examiner.

Participants then completed the following two tests, with the testing order determined using a randomized sealed-envelope method. To minimize fatigue effects, an interval of at least 2 days was provided between tests. Pulmonary gas exchange was measured on a breath-by-breath basis during both the r3MSST and CPET using an AE-310S metabolic cart (Minato Medical Science Co., Osaka, Japan), with participants wearing a respiratory gas analysis mask. Before measurements commenced each day, the gas analyzer was calibrated according to the manufacturer’s recommended procedures. The flow meter was calibrated three times using a 2-L calibration syringe after gas analyzer calibration. The acquired breath-by-breath data were processed using a 4-s moving average, and 15-s averaged values were exported as CSV data. Peak $\dot{V}$O_2_ was defined as the highest 15-s averaged $\dot{V}$O_2_ value obtained during CPET.

① The Revised 3-Minute Seated Stepping Test (r3MSST)

The r3MSST was conducted according to the method described by Nakagawa et al. [10,11], with minor modifications. A 40-cm-high chair and a fixed walker with an adjustable string were used to standardize step height at 10 cm above the floor.

Participants sat on the front edge of the chair without contacting the backrest. After 3 min of seated rest, they performed stepping movements for 3 min, raising each thigh to contact the string while completing as many steps as possible and keeping at least one foot on the floor.

After the first trial was completed, participants remained seated for 10 min to permit recovery of vital signs and subjective fatigue before a second trial was performed under identical conditions.

The test was recorded using a digital video camera (HC-WX990M, Panasonic Co., Osaka, Japan) positioned in front of the participant; the face was excluded from the frame to preserve anonymity. Videos were recorded in 720p MP4 format with the camera positioned 1.8 m from the chair and adjusted to clearly capture thigh contact with the string. The measurement environment and participant posture are shown in Figure 1a,b. Step counts were determined from slow-motion video playback and defined as the number of times the thigh contacted the string attached to the walker. For the primary correlation and prediction analyses, the higher step count of the two trials was used as the representative r3MSST performance value. Both trials were used for the within-session repeatability analysis. To examine the robustness of this scoring rule, sensitivity analyses were performed using Trial 1, Trial 2, the mean of the two trials, and the maximum of the two trials. Two licensed physical therapists independently counted the steps, and discrepancies were resolved by joint video review. Slow-motion video recording and independent counting by two raters were used to maximize the accuracy of step-count measurement in the research setting and were not intended as requirements for routine clinical administration of the r3MSST.

② Cardiopulmonary Exercise Testing (CPET)

CPET was performed as a symptom-limited maximal exercise test on a cycle ergometer (Corival CPET, Lode B.V., Groningen, The Netherlands). Participants completed a 2-min warm-up at 20 W, followed by a ramp protocol with 20 W/min increments. Pedaling cadence was maintained at 50–60 rpm.

A 12-lead electrocardiogram was continuously monitored during exercise, and blood pressure was automatically measured at 1-min intervals for safety monitoring. The test was continued until participants reached symptom limitation and was terminated if they were unable to continue because of severe dyspnea or leg fatigue, were unable to maintain the target pedaling cadence of 50–60 rpm, or developed a serious arrhythmia. After termination, participants completed a 3-min cool-down followed by 10 min of seated rest.

The following parameters were measured using the gas analysis system: peak minute ventilation (peak $\dot{V}$E), peak oxygen uptake (peak $\dot{V}$O_2_), peak carbon dioxide output (peak $\dot{V}$CO_2_), peak heart rate (peak HR), rate-pressure product (RPP), and peak oxygen pulse (peak $\dot{V}$O_2_/HR). Heart rate (HR) was measured before and after exercise. Blood pressure values obtained before and after exercise were recorded for data analysis. Dyspnea and lower-limb fatigue were assessed before exercise and at exercise termination using the Borg 6–20 rating of perceived exertion scale. Peak-exercise metabolic equivalents (METs) were calculated for CPET.

### 2.5. Statistical Analysis

To examine the relationship between the r3MSST and CPET, the normality of each variable was assessed using the Shapiro–Wilk test. Additionally, histograms and Q–Q plots were visually inspected to support the assessment. Because several variables showed non-normal distributions, Spearman’s rank correlation coefficients with 95% confidence intervals (CIs) were used to assess the associations between r3MSST- and CPET-derived variables. Confidence intervals for Spearman’s correlation coefficients were estimated using the Fieller, Hartley, and Pearson method implemented in SPSS. Absolute peak $\dot{V}$O_2_ (mL/min) was used as the criterion measure because the main objective was to examine the correspondence between directly measured oxygen uptake during the r3MSST and CPET and to develop a prediction equation for absolute CPET-derived peak $\dot{V}$O_2_. The association between r3MSST- and CPET-derived absolute peak $\dot{V}$O_2_ was designated as the primary association analysis. Correlations involving other physiological variables and paired comparisons between the two tests were considered secondary or exploratory analyses and were interpreted with emphasis on effect estimates and 95% CIs rather than statistical significance thresholds alone. Because absolute peak $\dot{V}$O_2_ is influenced by sex and body size, sensitivity analyses were additionally performed using body-mass-normalized peak $\dot{V}$O_2_ (mL/kg/min). Spearman’s rank correlation coefficient between r3MSST- and CPET-derived body-mass-normalized peak $\dot{V}$O_2_ was calculated, and exploratory sex-stratified correlations were also examined. In addition, the association between r3MSST step count and CPET-derived body-mass-normalized peak $\dot{V}$O_2_ was assessed. To further examine whether the association between absolute peak $\dot{V}$O_2_ measured during the r3MSST and CPET was independent of sex and body size, a sensitivity multiple regression analysis was performed with CPET-derived absolute peak $\dot{V}$O_2_ as the dependent variable and r3MSST-derived absolute peak $\dot{V}$O_2_, sex, and body mass index (BMI) as independent variables. Differences between the two tests were analyzed using the Wilcoxon signed-rank test. For variables assessed both before and after exercise (systolic blood pressure [SBP], diastolic blood pressure [DBP], dyspnea, and lower-limb fatigue), changes from baseline were calculated as post-exercise minus pre-exercise values. The normality of the paired differences in these changes between the two tests was assessed using the Shapiro–Wilk test. Between-test differences in changes from baseline were analyzed using paired-samples *t*-tests when the paired differences were normally distributed and Wilcoxon signed-rank tests when they were not normally distributed. Effect sizes were expressed as Cohen’s d for paired-samples *t*-tests and as r for Wilcoxon signed-rank tests. Effect sizes (r) were calculated as Z/√*N*, where *N* represents the number of paired observations. Within-session repeatability was evaluated using a two-way mixed-effects, absolute-agreement, single-measure intraclass correlation coefficient (ICC) and Bland–Altman analysis to assess systematic bias and limits of agreement. Proportional bias was assessed using Pearson’s correlation coefficient between the mean and the difference of the two trials. The standard error of measurement (SEM) was calculated as SD × √(1 − ICC), and the minimal detectable change at the 95% confidence level (MDC_95_) was calculated as SEM × 1.96 × √2.

To develop a prediction equation for CPET-derived peak $\dot{V}$O_2_, candidate predictors were initially evaluated using stepwise multiple regression analysis, with CPET-derived peak $\dot{V}$O_2_ as the dependent variable. Candidate predictors included sex, age, BMI, r3MSST step count, pre- and post-exercise HRs, pre- and post-exercise systolic and diastolic blood pressures, pre- and post-exercise ratings of dyspnea and lower-limb fatigue, and iliopsoas and quadriceps muscle strength. Variables were entered into and removed from the stepwise model using significance criteria of *p* < 0.05 and *p* > 0.10, respectively. The stepwise procedure identified sex, age, BMI, pre-exercise heart rate, and r3MSST step count as predictors. These five variables were subsequently entered simultaneously into a forced-entry multiple regression model to estimate the final prediction equation. Multicollinearity was assessed using variance inflation factors, and residual independence was evaluated using the Durbin–Watson statistic. Potentially influential observations were examined using Cook’s distance, leverage values, studentized residuals, and DFBETAs, which quantify the influence of individual observations on regression coefficients. Sensitivity analyses were performed by refitting the final model after individually excluding observations identified as potentially influential. Linearity and homoscedasticity were assessed by visual inspection of residual-versus-fitted and residual-versus-predictor plots, and homoscedasticity was additionally evaluated using the Breusch–Pagan test. Uncertainty around the regression coefficients was quantified using 95% CIs. The predictive model was internally validated using 2000 bootstrap resamples. In each bootstrap resample, the complete stepwise variable-selection procedure was repeated using the same candidate predictors and entry and removal criteria as in the original dataset. Model performance was assessed using the coefficient of determination (R^2^), root mean square error (RMSE), and calibration slope. The model developed in each bootstrap sample was evaluated both in the bootstrap sample and in the original dataset to estimate optimism. Optimism-corrected performance measures were calculated by subtracting the mean optimism across the 2000 bootstrap resamples from the apparent performance in the original dataset. To assess the incremental predictive contribution of the r3MSST, a base model including sex, age, BMI, and pre-exercise HR was compared with an extended model that additionally included r3MSST step count. The incremental contribution of r3MSST step count was evaluated using changes in R^2^ and RMSE. The statistical significance of the improvement in model fit was assessed using an F-test for nested models. Optimism-corrected changes in R^2^ and RMSE were estimated using 2000 bootstrap resamples.

Statistical analyses were performed using IBM SPSS Statistics version 30.0 (IBM Corp., Armonk, NY, USA). Effect size calculations for the Wilcoxon signed-rank tests and bootstrap internal validation of the predictive model were performed using R version 4.6.1 (R Foundation for Statistical Computing, Vienna, Austria). The level of statistical significance was set at *p* < 0.05.

## 3. Results

In total, 57 healthy young adults (28 men and 29 women) were included in the final analysis. Participant characteristics are presented in Table 1.

Peak $\dot{V}$O_2_ measured during the r3MSST showed a strong positive association with peak $\dot{V}$O_2_ measured during CPET (r_s_ = 0.725, 95% CI: 0.567–0.831, *p* < 0.001). Similarly, peak $\dot{V}$O_2_/HR measured during the r3MSST showed a strong positive association with peak $\dot{V}$O_2_/HR measured during CPET (r_s_ = 0.692, 95% CI: 0.521–0.810, *p* < 0.001) (Table 2, Figure 2 and Figure 3). In contrast, the estimated association between r3MSST step count and CPET-derived peak $\dot{V}$O_2_ was weak (r_s_ = 0.077, 95% CI: −0.195 to 0.338), with the CI compatible with both negative and positive associations (Figure 4). In sensitivity analyses using body-mass-normalized peak $\dot{V}$O_2_, the association between r3MSST- and CPET-derived peak $\dot{V}$O_2_ was attenuated but remained moderate in magnitude (r_s_ = 0.462, 95% CI: 0.222–0.650, *p* < 0.001). In exploratory sex-stratified analyses of body-mass-normalized peak $\dot{V}$O_2_, the estimated association was stronger in women (r_s_ = 0.400, 95% CI: 0.028–0.675, n = 29) than in men (r_s_ = 0.129, 95% CI: −0.268 to 0.488, n = 28), although the CIs were wide and overlapping. The association between r3MSST step count and CPET-derived body-mass-normalized peak $\dot{V}$O_2_ was weak (r_s_ = 0.191, 95% CI: −0.081 to 0.436, *p* = 0.155). In an additional sensitivity regression analysis adjusting for sex and BMI, the estimated association between r3MSST-derived and CPET-derived absolute peak $\dot{V}$O_2_ was positive but imprecise (B = 0.454, 95% CI: −0.064 to 0.972, *p* = 0.085; R^2^ = 0.735, adjusted R^2^ = 0.720).

Secondary and exploratory correlation analyses showed positive associations of varying magnitude between the r3MSST and CPET. The estimates were moderate to strong for peak $\dot{V}$CO_2_ (r_s_ = 0.641, 95% CI: 0.451–0.776), peak METs (r_s_ = 0.462, 95% CI: 0.222–0.650), peak $\dot{V}$E (r_s_ = 0.396, 95% CI: 0.143–0.600), and peak HR (r_s_ = 0.373, 95% CI: 0.117–0.583). Positive associations were also observed for pre- and post-exercise HR and blood pressure measures, although the magnitude and precision of the estimates varied (Table 2). In particular, the post-exercise SBP correlation was weak and imprecisely estimated (r_s_ = 0.263, 95% CI −0.006 to 0.496), with the CI including zero despite a two-sided *p* value of 0.048. For RPP at the peak exercise and peak respiratory rate, the estimated correlations were small (r_s_ = 0.205 and 0.224, respectively), and their 95% CIs included zero. For subjective measures, pre-exercise dyspnea showed a positive association (r_s_ = 0.392, 95% CI: 0.139–0.597), whereas the estimates for post-exercise dyspnea and pre- and post-exercise lower-limb fatigue were small and imprecise, with 95% CIs including zero.

Comparisons between CPET and r3MSST outcomes demonstrated significant differences for several physiological variables (Table 3).

Exercise capacity and metabolic parameters, including peak $\dot{V}$O_2_, peak $\dot{V}$O_2_/HR, peak $\dot{V}$CO_2_, peak $\dot{V}$E, and peak METs, were significantly higher during CPET (all *p* < 0.001), with large effect sizes (r = 0.870 for each variable). Peak HR and RPP were also significantly greater during CPET (both *p* < 0.001), with large effect sizes (r = 0.870 and 0.863, respectively). Peak respiratory rate was also significantly higher during CPET (*p* < 0.001, r = 0.858). For variables measured both before and after exercise, between-test comparisons were based on changes from baseline (post-exercise minus pre-exercise). The change in SBP did not differ significantly between the r3MSST and CPET (18.05 ± 10.65 vs. 16.16 ± 17.69 mmHg, respectively; *p* = 0.525). In contrast, the change in DBP differed significantly between the tests (2.0 [−1.5 to 11.5] vs. −6.0 [−12.5 to 5.5] mmHg, respectively; *p* < 0.001). Changes in dyspnea and lower-limb fatigue were significantly greater during CPET than during the r3MSST (dyspnea: 8.0 [7.0–9.5] vs. 3.0 [2.0–5.0], *p* < 0.001; lower-limb fatigue: 9.75 ± 2.18 vs. 5.30 ± 2.13, *p* < 0.001).

Multiple regression analysis (Table 4) selected five predictors of CPET-derived peak $\dot{V}$O_2_: sex, age, BMI, pre-exercise heart rate, and r3MSST step count. Iliopsoas and quadriceps muscle strength were included among the candidate predictors but were not retained by the stepwise selection procedure. All five variables were statistically significant in the subsequent forced-entry model. The final model was statistically significant (F(5,51) = 61.610, *p* < 0.001), with an R^2^ of 0.858 and an adjusted R^2^ of 0.844. Standardized regression coefficients were as follows: sex (β = 0.759, *p* < 0.001), age (β = −0.240, *p* < 0.001), BMI (β = 0.190, *p* = 0.002), pre-exercise heart rate (β = −0.295, *p* < 0.001), and r3MSST step count (β = 0.120, *p* = 0.035). The corresponding unstandardized regression coefficients (95% CIs) were 862.196 (738.023–986.368) for sex, −104.075 (−151.549 to −56.602) for age, 42.022 (16.286–67.758) for BMI, −10.156 (−14.007 to −6.306) for pre-exercise HR, and 0.402 (0.030–0.775) for r3MSST step count. Variance inflation factors ranged from 1.064 to 1.209, indicating no evidence of multicollinearity. The Durbin-Watson statistic was 2.23, supporting the independence of residuals. Visual inspection of residual-versus-fitted and residual-versus-predictor plots did not indicate marked departures from linearity or a clear pattern of heteroscedasticity. The Breusch–Pagan test was not statistically significant (BP = 5.623, df = 5, *p* = 0.345), providing no evidence of heteroscedasticity. Examination of Cook’s distance, leverage, studentized residuals, and DFBETAs identified several potentially influential observations; however, sensitivity analyses in which these observations were individually excluded produced only modest changes in model performance and regression coefficients, without materially altering the overall findings. Based on these findings, the prediction equation for CPET-derived peak $\dot{V}$O_2_ was as follows: peak $\dot{V}$O_2_ (CPET) = 3177.935 + 862.196 × sex − 104.075 × age + 42.022 × BMI − 10.156 × pre-exercise heart rate + 0.402 × r3MSST step count, where sex was coded as male = 1 and female = 0. Because the age coefficient was estimated within the narrow age range of 18–23 years, this preliminary prediction model should not be applied outside the development population without external validation. The apparent RMSE of the prediction model was 214.1 mL/min. Bootstrap internal validation, in which the complete stepwise variable-selection procedure was repeated within each of 2000 resamples, yielded optimism-corrected values of 0.788 for R^2^, 269.4 mL/min for RMSE, and 0.960 for the calibration slope.

To assess the incremental predictive contribution of the r3MSST, the extended model was compared with a base model including sex, age, BMI, and pre-exercise HR. The base model had an R^2^ of 0.845 and an RMSE of 223.8 mL/min, whereas the extended model had an R^2^ of 0.858 and an RMSE of 214.1 mL/min. Thus, adding r3MSST step count increased R^2^ by 0.013 and reduced RMSE by 9.7 mL/min. The improvement in model fit was statistically significant (F(1,51) = 4.707, *p* = 0.035). After correction for optimism using 2000 bootstrap resamples, the incremental improvement was attenuated, with an optimism-corrected ΔR^2^ of 0.009 and an optimism-corrected reduction in RMSE of 5.8 mL/min. Sensitivity analyses using alternative r3MSST scoring rules yielded similar findings. The correlations between r3MSST step count and CPET-derived peak $\dot{V}$O_2_ were weak across Trial 1 (r_s_ = 0.022, *p* = 0.872), Trial 2 (r_s_ = 0.129, *p* = 0.338), the mean of the two trials (r_s_ = 0.108, *p* = 0.424), and the maximum of the two trials (r_s_ = 0.077, *p* = 0.567). Similarly, prediction-model performance was nearly unchanged across these scoring rules, with R^2^ values of 0.857, 0.858, 0.859, and 0.858, respectively. In exploratory sex-stratified analyses, the estimated association between r3MSST step count and CPET-derived peak $\dot{V}$O_2_ was negligible in both women (r_s_ = 0.003, *p* = 0.989, n = 29) and men (r_s_ = 0.032, *p* = 0.870, n = 28). Given the small sample sizes within each sex, sex-specific multivariable prediction models were not developed.

The mean step count was 619.4 ± 157.4 steps for Trial 1 and 702.2 ± 174.9 steps for Trial 2. Bland–Altman analysis (Figure 5) showed a mean difference of −82.8 steps (Trial 1 minus Trial 2), with 95% limits of agreement from −250.5 to 84.9 steps. Most points lay within the limits of agreement. The correlation between the mean and the difference of the two trials was not statistically significant (r = −0.211, *p* = 0.115, 95% CI: −0.447 to 0.053). No clear proportional association was detected, although the estimate was imprecise. The ICC was 0.774 (95% CI: 0.231–0.911, *p* < 0.001). The SEM was 79.1 steps, and the MDC_95_ was 219.2 steps. Inter-rater reliability for step counting was excellent for both trials, with single-measure, absolute-agreement ICCs of 0.999998 (95% CI: 0.999997–0.999999) for Trial 1 and 0.999993 (95% CI: 0.999989–0.999996) for Trial 2.

## 4. Discussion

This study examined the physiological associations and within-session repeatability of the r3MSST and explored the prediction of peak $\dot{V}$O_2_ in healthy young adults. Peak $\dot{V}$O_2_ measured during the r3MSST showed a strong positive association with peak $\dot{V}$O_2_ measured during CPET. However, the within-session repeatability of the r3MSST was uncertain, with substantial measurement variability and a systematic between-trial difference.

### 4.1. Relationship with CPET

In this study, peak $\dot{V}$O_2_ measured during the r3MSST showed a strong positive association with peak $\dot{V}$O_2_ measured during CPET (r_s_ = 0.725, 95% CI: 0.567–0.831, *p* < 0.001). Peak $\dot{V}$O_2_ is widely accepted as the most robust measure of cardiorespiratory fitness and reflects the integrated responses of the pulmonary, cardiovascular, and skeletal muscle systems during exercise [2]. The findings indicate that physiological responses during the r3MSST covary with CPET-derived peak $\dot{V}$O_2_. However, this physiological association should be distinguished from the criterion validity of the r3MSST step count as a field-based outcome. In the present study, the estimated association between r3MSST step count and CPET-derived peak $\dot{V}$O_2_ was weak (r_s_ = 0.077, 95% CI −0.195 to 0.338), with the CI compatible with both negative and positive associations. Thus, although physiological responses measured during the r3MSST were associated with corresponding CPET-derived measures, the criterion validity of step count alone for estimating peak $\dot{V}$O_2_ was not sufficiently supported by the present data.

The sensitivity analyses further indicated that the association between r3MSST- and CPET-derived peak $\dot{V}$O_2_ was influenced, at least in part, by sex and body size. When peak $\dot{V}$O_2_ was normalized to body mass, the association was attenuated from r_s_ = 0.725 to r_s_ = 0.462 (95% CI: 0.222–0.650), indicating a more moderate association in the overall sample. In exploratory sex-stratified analyses of body-mass-normalized peak $\dot{V}$O_2_, the estimated association was stronger in women (r_s_ = 0.400, 95% CI: 0.028–0.675) than in men (r_s_ = 0.129, 95% CI −0.268 to 0.488); however, the CIs were wide and overlapping, and these subgroup findings should be interpreted cautiously because of the small sample sizes. Furthermore, the association between r3MSST step count and CPET-derived body-mass-normalized peak $\dot{V}$O_2_ was weak (r_s_ = 0.191, 95% CI −0.081 to 0.436). In addition, after adjustment for sex and BMI, the estimated association between r3MSST-derived and CPET-derived absolute peak $\dot{V}$O_2_ was positive but imprecise (B = 0.454, 95% CI: −0.064 to 0.972). Collectively, these findings suggest that shared anthropometric and sex-related differences may partly contribute to the association observed between absolute peak $\dot{V}$O_2_ values obtained during the two tests. Therefore, the association based on absolute peak $\dot{V}$O_2_ should not be interpreted as evidence that r3MSST performance itself provides strong information regarding exercise capacity independent of sex and body size. Positive associations of varying magnitude were also observed between corresponding r3MSST- and CPET-derived physiological variables, including peak $\dot{V}$O_2_/HR, peak $\dot{V}$CO_2_, peak METs, peak $\dot{V}$E, and peak HR. These findings provide additional evidence that physiological responses during the r3MSST are associated with corresponding CPET-derived measures, but they should not be interpreted as establishing the criterion validity of r3MSST step count as a field-based measure of exercise capacity. Previous studies have reported that peak $\dot{V}$O_2_ obtained during the 3MSST is approximately 50% of that measured during CPET [11], indicating that the 3MSST reflects submaximal exercise intensity. Furthermore, these findings are consistent with previous studies demonstrating associations between performance on submaximal field exercise tests, such as the 6MWT, and peak $\dot{V}$O_2_ [4]. Recent studies have also developed peak $\dot{V}$O_2_ or $\dot{V}$O_2_max prediction equations using step-test performance and physiological variables. Selland et al. developed a generalized peak $\dot{V}$O_2_ prediction equation based on the YMCA step test using age, sex, abdominal circumference, and exercise HR, and validated the equation in an independent sample [12]. Matsuo et al. also developed and cross-validated a $\dot{V}$O_2_max prediction model using HR responses during and after a 3-min stepping exercise [13]. These findings support the use of step-test-derived physiological responses and participant characteristics for estimating cardiorespiratory fitness and provide relevant context for the present r3MSST prediction model. Similarly, Rowley et al. [5] demonstrated that simple walking and stepping field tests could be used to estimate $\dot{V}$O_2_max in healthy adults, supporting the potential utility of field-based assessments of exercise capacity. Moreover, Nakagawa et al. [10] reported a significant correlation between 3MSST step counts and 6MWT walking distance (r_s_ = 0.74) in patients with cardiovascular disease, further supporting the ability of the 3MSST to reflect exercise capacity.

Multiple regression analysis selected sex, age, BMI, pre-exercise HR, and r3MSST step count as predictors of CPET-derived peak $\dot{V}$O_2_. The strong association of sex with peak $\dot{V}$O_2_ may partly reflect physiological differences between men and women, including differences in muscle mass and hemoglobin concentration that influence oxygen transport capacity [14,15]. Age and BMI are established factors associated with cardiorespiratory fitness and may contribute to inter-individual differences in peak $\dot{V}$O_2_. In exploratory sex-stratified analyses, however, r3MSST step count was not significantly associated with CPET-derived peak $\dot{V}$O_2_ in either women or men. These findings should be interpreted cautiously because of the small sample sizes within each sex and do not establish the absence of a sex-specific association. Accordingly, adequately powered studies are needed to further examine potential sex differences in the relationship between r3MSST performance and CPET-derived peak $\dot{V}$O_2_ and to determine whether sex-specific prediction models are warranted.

Pre-exercise HR was negatively associated with CPET-derived peak $\dot{V}$O_2_ in the present model. This finding is consistent with previous population-based studies demonstrating an inverse association between resting HR and cardiorespiratory fitness, including peak $\dot{V}$O_2_ or estimated $\dot{V}$O_2_max [16,17]. Higher resting HR has been associated with lower cardiorespiratory fitness both cross-sectionally and longitudinally, suggesting that resting HR may provide complementary physiological information related to cardiorespiratory fitness [16,17]. However, because resting HR is influenced by factors such as physical activity and adiposity, its role in the present prediction equation should be interpreted as predictive rather than causal.

Step count on the r3MSST also contributed to the multivariable prediction model for CPET-derived peak $\dot{V}$O_2_ when considered together with the other predictors. However, this model-based contribution should be distinguished from the weak univariable association between r3MSST step count and CPET-derived peak $\dot{V}$O_2_ and does not, by itself, establish the predictive utility of step count. Moreover, the incremental predictive contribution of r3MSST step count beyond readily available participant characteristics was modest. Compared with the base model including sex, age, BMI, and pre-exercise HR, adding r3MSST step count increased the apparent R^2^ by 0.013 and reduced the apparent RMSE by 9.7 mL/min. After correction for optimism using 2000 bootstrap resamples, these improvements were attenuated to a ΔR^2^ of 0.009 and a reduction in RMSE of 5.8 mL/min. Thus, although the addition of r3MSST step count produced a small improvement in model performance, its incremental predictive contribution beyond the variables included in the base model was limited and requires confirmation in independent samples. The prediction model showed high apparent performance (R^2^ = 0.858); however, given the relatively small sample size (n = 57) and the inclusion of five predictors, overfitting remains a concern. Bootstrap internal validation indicated some optimism in the apparent model performance (optimism-corrected R^2^ = 0.788), underscoring the need for external validation in independent populations before the prediction equation can be applied more broadly. Taken together, these findings provide preliminary evidence that physiological responses measured during the r3MSST are associated with CPET-derived measures of exercise capacity; the predictive utility of r3MSST step count and the broader practical applicability of the test require further validation.

### 4.2. Comparison of Physiological Responses with CPET

The physiological responses observed during the r3MSST were generally lower than those observed during CPET, reflecting the lower exercise intensity of the r3MSST. These differences are likely attributable to the symptom-limited maximal nature of CPET compared with the submaximal nature of the r3MSST. This interpretation is consistent with previous findings showing that peak $\dot{V}$O_2_ measured during the 3MSST reaches approximately 50% of that measured during CPET [11], further supporting the submaximal nature of the r3MSST.

Although the r3MSST does not directly assess maximal exercise capacity, the observed associations with CPET-derived variables suggest that it may provide useful information regarding submaximal exercise capacity. Blood pressure responses showed less consistent differences between the two tests, which may reflect the influence of factors other than exercise intensity, including autonomic nervous system activity and peripheral vascular function [18,19,20].

Subjective responses, including post-exercise dyspnea and lower limb fatigue, were greater during CPET, consistent with its higher physiological demand. Taken together, the lower physiological and subjective responses observed during the r3MSST support its characterization as a submaximal field exercise test.

### 4.3. Within-Session Repeatability

The ICC was 0.774 (95% CI: 0.231–0.911). Although the point estimate suggested moderate-to-good relative within-session repeatability, the wide CI indicates substantial uncertainty in the precision of this estimate. The SEM was 79.1 steps, and the MDC_95_ was 219.2 steps, indicating substantial absolute measurement error; thus, a relatively large change in step count would be required to exceed measurement variability.

The mean step count increased from 619.4 ± 157.4 steps in Trial 1 to 702.2 ± 174.9 steps in Trial 2. Consistent with this increase, Bland–Altman analysis showed a mean difference of −82.8 steps (Trial 1 minus Trial 2), with 95% limits of agreement ranging from −250.5 to 84.9 steps. Although no clear proportional association was detected, the estimate was imprecise, and the higher step count in Trial 2 indicates a systematic trial effect that should not be interpreted solely as random measurement variability. This systematic increase may reflect familiarization, learning, pacing, or other order-related effects. Because the present study was not designed to distinguish among these potential explanations, their relative contributions cannot be determined. These findings suggest that the potential need for familiarization or repeated testing should be considered when using the r3MSST.

Sensitivity analyses showed that the main findings were not materially affected by the choice of scoring rule. The association between r3MSST step count and CPET-derived peak $\dot{V}$O_2_ remained weak across Trial 1, Trial 2, the mean of the two trials, and the maximum of the two trials, and prediction-model performance was nearly unchanged across these scoring approaches. Nevertheless, given the systematic increase in step count from Trial 1 to Trial 2, the present findings do not establish that selecting the maximum of two trials is the optimal scoring protocol for clinical use. Further studies should determine whether a single trial, a familiarization trial followed by a measured trial, the mean of repeated trials, or the maximum of repeated trials provides the most reliable and clinically appropriate protocol.

Taken together, the wide 95% CI of the ICC, the relatively wide limits of agreement, and the large MDC_95_ suggest that changes in step count should be interpreted with caution at the individual level. Further studies are needed to determine the optimal familiarization procedure, assess between-day test–retest reliability, and define clinically meaningful thresholds for change.

### 4.4. Practical Implications

The r3MSST requires only a chair, minimal space, and a short testing period, which are operational advantages of the test. However, the present study does not establish that the r3MSST is sufficiently reliable, precise, externally valid, or practically applicable for routine individual-level assessment. In particular, the uncertainty in within-session repeatability, the systematic difference between repeated trials, the limited incremental predictive contribution of step count, and the lack of external validation preclude conclusions regarding routine clinical use. Therefore, the present findings should be regarded as preliminary and should not be extrapolated to older adults or clinical populations; the directions for future research are outlined in the conclusions.

## 5. Limitations

This study has several limitations. First, only healthy young adults with a relatively narrow age distribution were included; therefore, the present findings should be interpreted as applying specifically to this population and should not be generalized to older adults or clinical populations. The clinical applicability of the r3MSST cannot be established from the present study. Further studies are required to validate the r3MSST in older adults and patients with cardiovascular or respiratory diseases.

Second, repeatability was assessed within the same session rather than across separate days. Therefore, the present findings should be interpreted as evidence regarding within-session repeatability rather than between-day test–retest reliability. The systematic increase in step count from Trial 1 to Trial 2 suggests a possible familiarization, learning, pacing, or order effect, although the present study was not designed to distinguish among these explanations. In addition, the higher step count of the two trials was used as the primary r3MSST score. Although sensitivity analyses using Trial 1, Trial 2, the mean, and the maximum yielded similar findings, the optimal scoring protocol has not been established. The ICC estimate was accompanied by a wide 95% CI, and the relatively wide limits of agreement and MDC_95_ further indicate substantial uncertainty and measurement variability at the individual level. Further studies should incorporate appropriate familiarization procedures and assess reliability across separate testing sessions.

Third, the prediction model was developed using stepwise variable selection in a relatively small sample (n = 57), with five predictors retained in the final model. This data-driven selection procedure may increase model instability and the risk of overfitting. Bootstrap internal validation with 2000 resamples, which repeated the complete variable-selection procedure, showed some reduction in predictive performance after optimism correction. Furthermore, the incremental predictive contribution of r3MSST step count beyond sex, age, BMI, and pre-exercise HR was small (optimism-corrected ΔR^2^ = 0.009 and reduction in RMSE = 5.8 mL/min). Thus, the additional predictive value of step count has not been firmly established. The prediction equation should therefore be regarded as preliminary and requires external validation in larger, independent populations before broader application.

Finally, the primary association analysis used absolute peak $\dot{V}$O_2_, which is influenced by sex and body size. Although sensitivity analyses using body-mass-normalized peak $\dot{V}$O_2_ and adjustment for sex and BMI were performed, the attenuation of the observed associations indicates that sex and body size may partly contribute to the relationship between r3MSST- and CPET-derived absolute peak $\dot{V}$O_2_. This should be considered when interpreting the criterion-related findings.

## 6. Conclusions

This study provides preliminary evidence that physiological responses measured during the r3MSST are associated with CPET-derived measures of exercise capacity in healthy young adults. In contrast, the association between r3MSST step count and CPET-derived peak $\dot{V}$O_2_ was weak. A prediction model incorporating participant characteristics, pre-exercise HR, and r3MSST step count showed promising internal performance; however, the incremental predictive contribution of step count was limited, and the model has not been externally validated. Within-session repeatability was uncertain and was accompanied by a systematic increase in step count between trials, suggesting a possible familiarization or order effect. Therefore, the present findings do not establish the reliability, predictive validity, or practical applicability of the r3MSST for routine individual-level use. Further studies should evaluate the criterion validity of the field-based step-count score, between-day reliability, appropriate familiarization and scoring procedures, external predictive validity, and performance in older adults and clinically relevant populations.

## Figures and Tables

**Figure 1:**
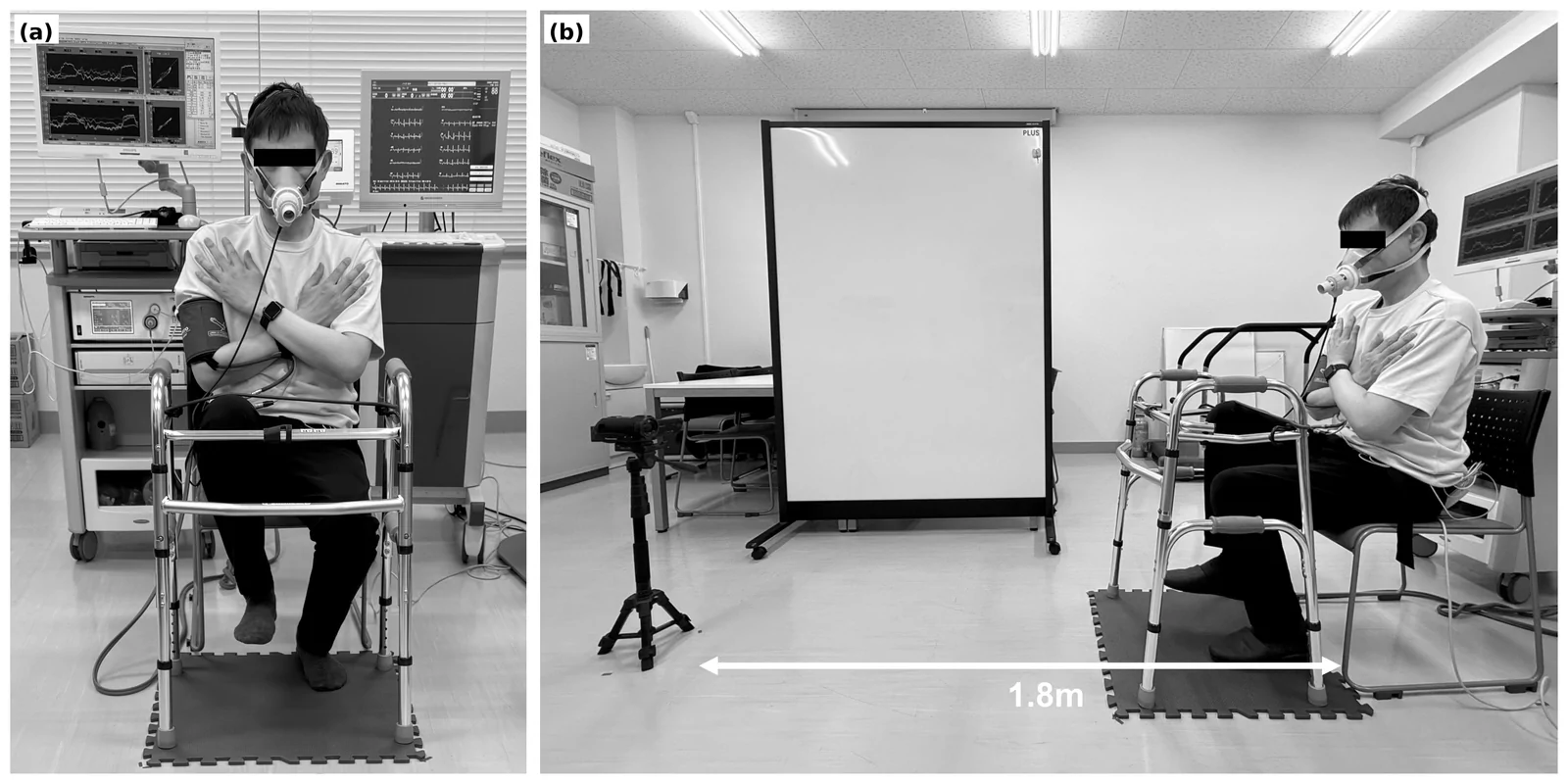
Measurement setup for the Revised 3-Minute Seated Stepping Test (r3MSST): (**a**) Participant posture during the test (front view); (**b**) Measurement environment and camera setup (side view).

**Figure 2:**
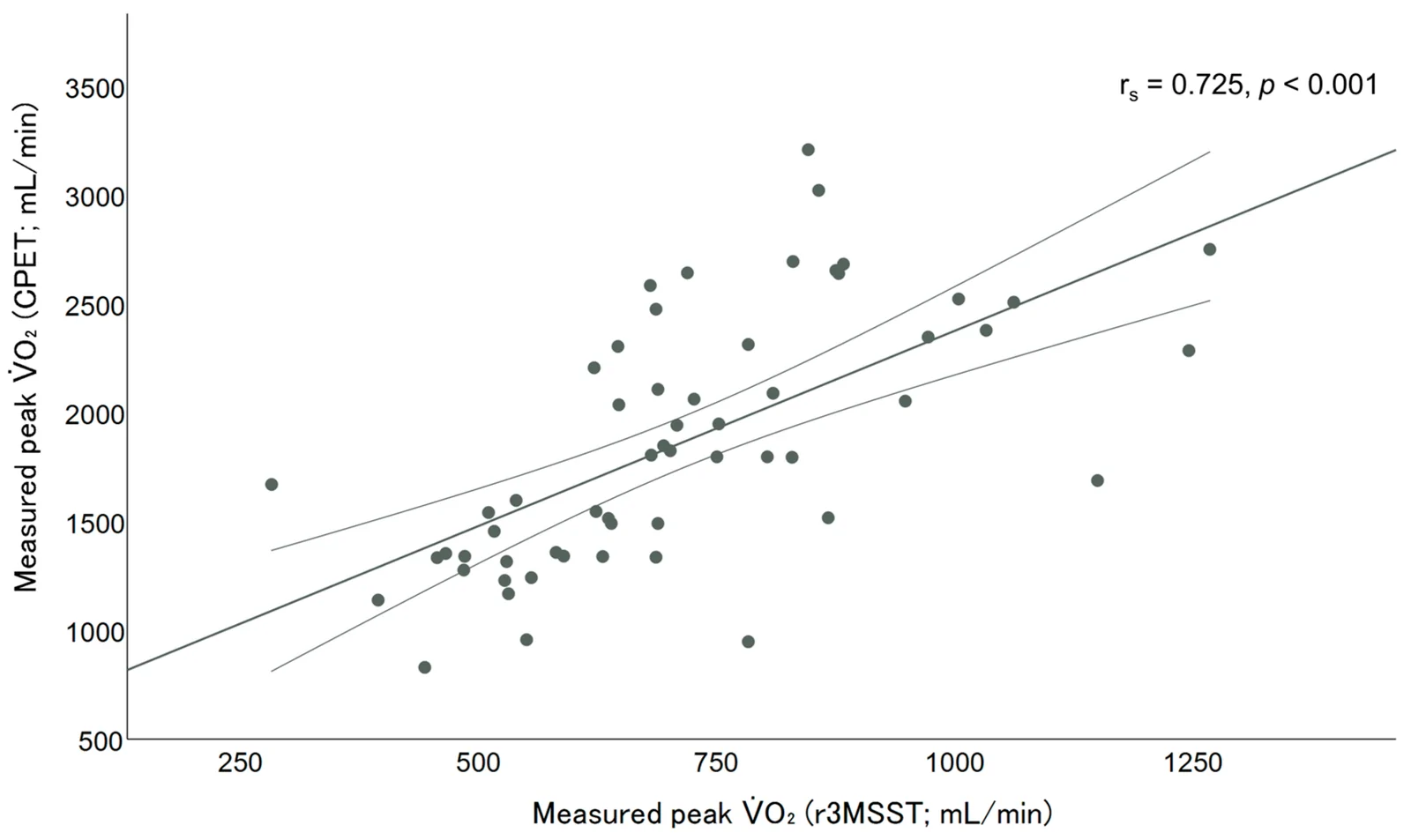
Relationship between measured peak $\dot{V}$O_2_ during CPET and measured peak $\dot{V}$O_2_ during the r3MSST. Each dot represents one participant. The solid line represents the regression line, and the two outer lines represent the 95% confidence interval.

**Figure 3:**
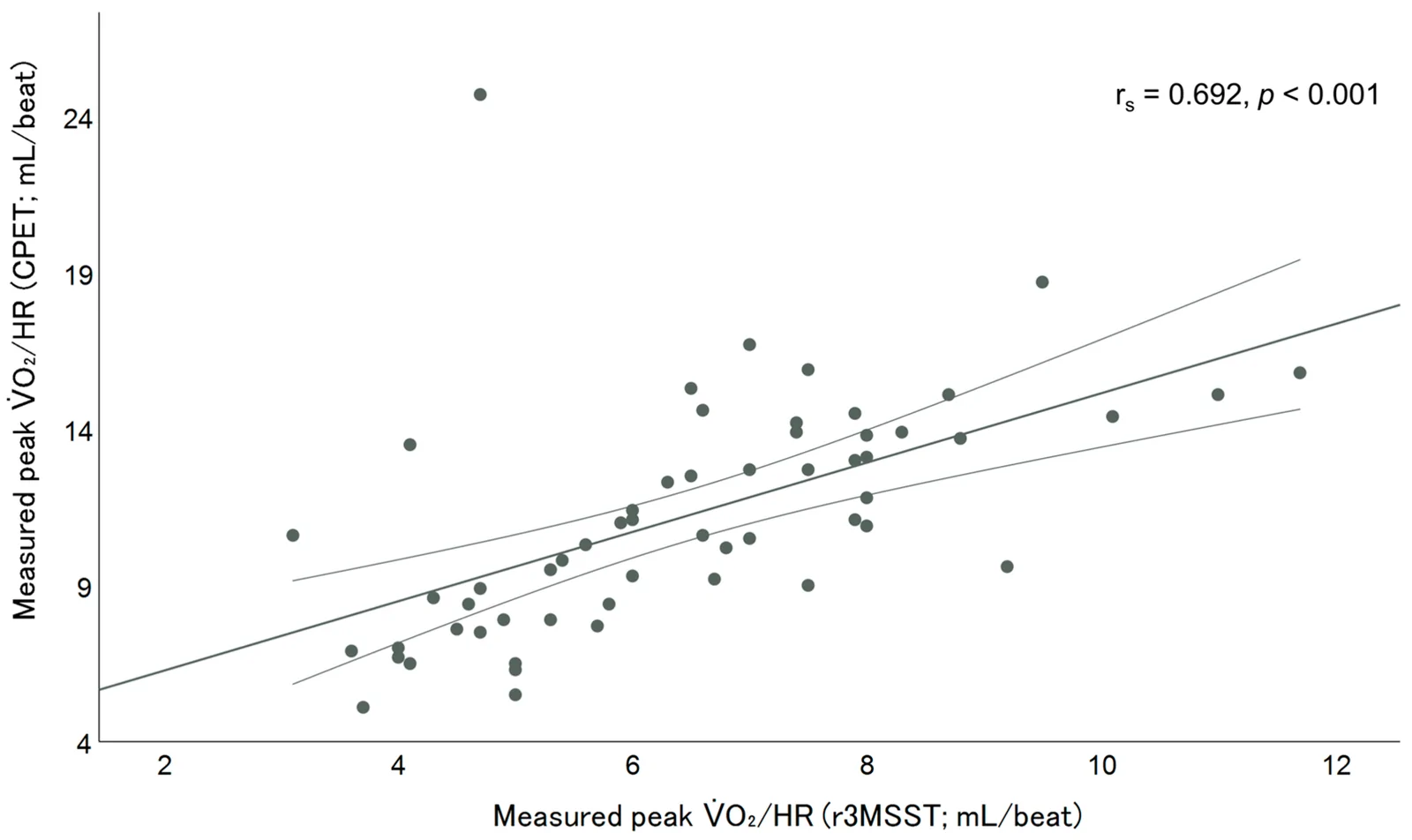
Relationship between measured peak $\dot{V}$O_2_/HR during CPET and measured peak $\dot{V}$O_2_/HR during the r3MSST. Each dot represents one participant. The solid line represents the regression line, and the two outer lines represent the 95% confidence interval.

**Figure 4:**
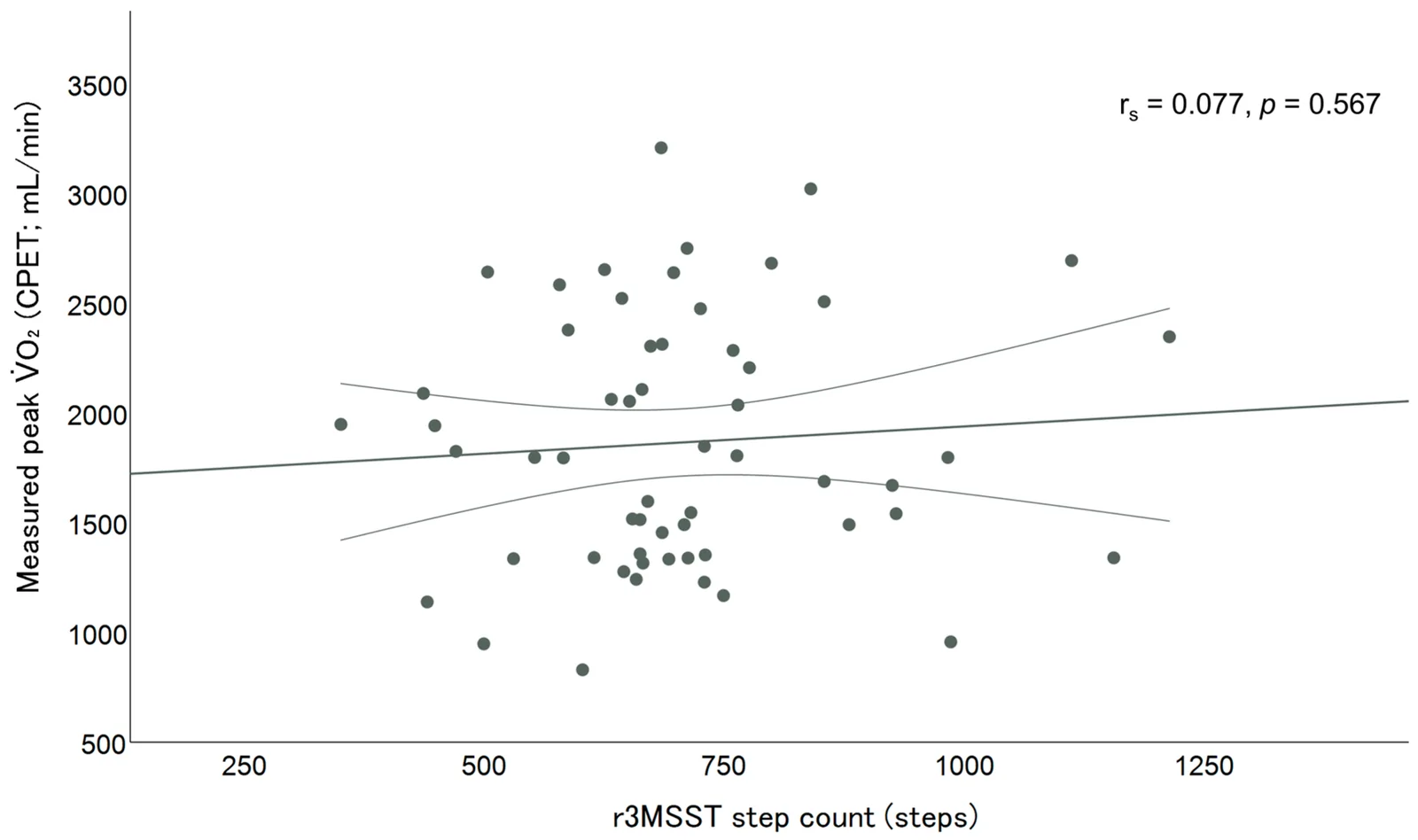
Relationship between r3MSST step count and peak $\dot{V}$O_2_ measured during CPET. Each dot represents one participant. The solid line represents the regression line, and the two outer lines represent the 95% confidence interval.

**Figure 5:**
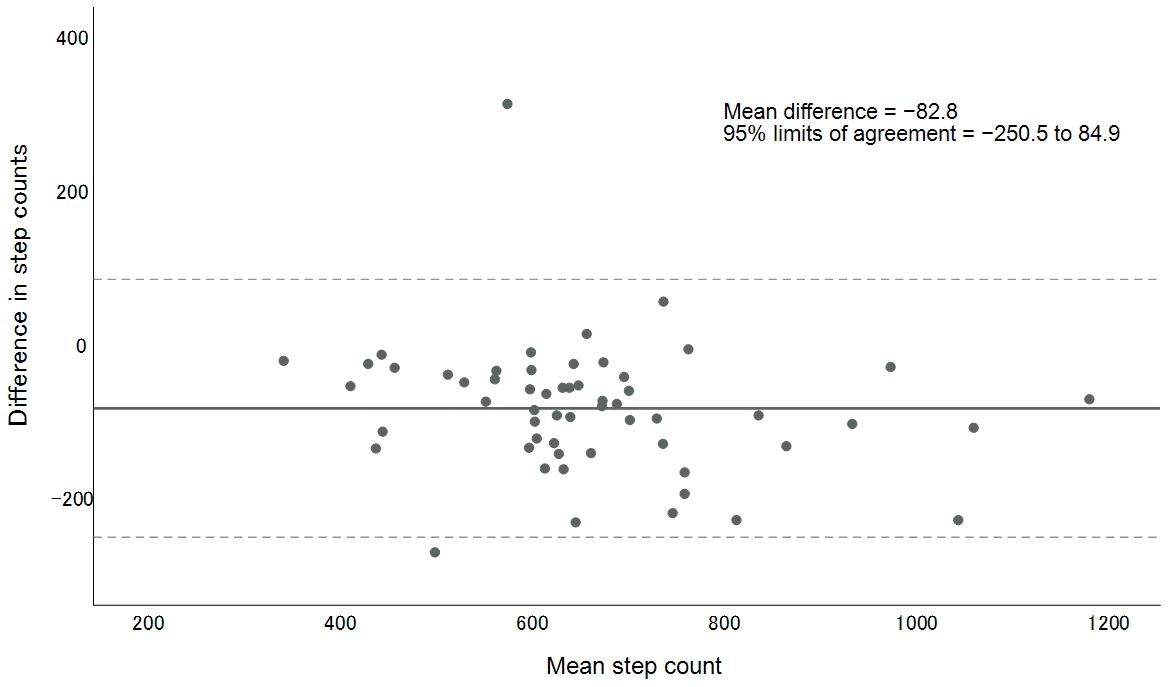
Bland–Altman plot of step counts between Trial 1 and Trial 2. Each dot represents one participant. The solid line denotes the mean difference, and the dashed lines denote the 95% limits of agreement. Differences were calculated as Trial 1 − Trial 2.

**Table 1:** Participant characteristics.

Variable	Value
Age (years)	20.2 ± 1.3
Sex, *n* (male/female)	28/29
Height (cm)	166.2 ± 10.4
Body mass (kg)	59.7 ± 9.0
BMI (kg/m^2^)	21.3 (20.0–22.9)
Right hip flexor (iliopsoas) muscle strength (N)	220.7 (178.5–264.8)
Left hip flexor (iliopsoas) muscle strength (N)	208.0 (163.8–264.8)
Right knee extensor (quadriceps) muscle strength (N)	323.7 (217.8–400.2)
Left knee extensor (quadriceps) muscle strength (N)	289.4 (210.9–422.8)

Note: Values are presented as mean ± standard deviation (SD) or median (interquartile range, IQR), according to data distribution. BMI = body mass index.

**Table 2:** Correlations between CPET and r3MSST variables.

Variable	r_s_	95% CI	*p*-Value
peak $\dot{V}$O_2_	0.725	0.567–0.831	<0.001
peak $\dot{V}$O_2_/HR (oxygen pulse)	0.692	0.521–0.810	<0.001
peak $\dot{V}$CO_2_	0.641	0.451–0.776	<0.001
peak METs	0.462	0.222–0.650	<0.001
peak $\dot{V}$E	0.396	0.143–0.600	0.002
peak HR	0.373	0.117–0.583	0.004
HR (pre-exercise)	0.634	0.441–0.771	<0.001
HR (post-exercise)	0.360	0.102–0.573	0.006
Systolic blood pressure (pre-exercise)	0.536	0.313–0.703	<0.001
Systolic blood pressure (post-exercise)	0.263	−0.006–0.496	0.048
Diastolic blood pressure (pre-exercise)	0.550	0.331–0.713	<0.001
Diastolic blood pressure (post-exercise)	0.273	0.005–0.504	0.040
Dyspnea (pre-exercise)	0.392	0.139–0.597	0.003
Dyspnea (post-exercise)	0.206	−0.006–0.449	0.125
Lower-limb fatigue (pre-exercise)	0.173	−0.100–0.421	0.199
Lower-limb fatigue (post-exercise)	0.244	−0.026–0.480	0.068
Rate-pressure product at peak exercise	0.205	−0.067–0.448	0.127
peak respiratory rate (breaths/min)	0.224	−0.047–0.464	0.094

Note: r3MSST = Revised 3-Minute Seated Stepping Test; r_s_ = Spearman’s rank correlation coefficient. CPET = cardiopulmonary exercise testing; peak $\dot{V}$O_2_ = peak oxygen uptake; HR = heart rate; $\dot{V}$E = minute ventilation; peak $\dot{V}$CO_2_ = peak carbon dioxide output; METs = metabolic equivalents. Dyspnea and lower-limb fatigue were assessed using the Borg 6–20 rating of perceived exertion scale.

**Table 3:** Comparison of Outcomes Between the r3MSST and CPET.

Variable	r3MSST	CPET	Effect Size	*p*-Value
SBP (pre-exercise)	113 (103–123)	116 (105–126)	—	—
SBP (post-exercise)	131 (121–143)	131 (124–140)	—	—
Change in SBP (mmHg)	18.05 ± 10.65	16.16 ± 17.69	0.085	0.525
DBP (pre-exercise)	80 (75–85)	77 (71–87)	—	—
DBP (post-exercise)	84 (76–91)	74 (68–84)	—	—
Change in DBP (mmHg)	2.0 (−1.5–11.5)	−6.0 (−12.5–5.5)	0.479	<0.001
Dyspnea (pre-exercise)	7 (7–8)	7 (7–7)	—	—
Dyspnea (post-exercise)	11 (9–13)	15 (15–17)	—	—
Change in dyspnea	3.0 (2.0–5.0)	8.0 (7.0–9.5)	0.845	<0.001
Lower-limb fatigue (pre-exercise)	7 (7–9)	7 (7–7)	—	—
Lower-limb fatigue (post-exercise)	13 (12–15)	17 (15–18)	—	—
Change in lower-limb fatigue	5.30 ± 2.13	9.75 ± 2.18	1.757	<0.001
peak HR (beats/min)	115 (106–126)	181 (172–189)	0.870	<0.001
peak $\dot{V}$O_2_ (mL/min)	691 (558–833)	1797 (1353–2313)	0.870	<0.001
Rate-pressure product	14,259 (11,781–16,128)	22,880 (21,484–25,520)	0.863	<0.001
peak $\dot{V}$O_2_/HR (mL/beat)	6.5 (5.0–7.9)	10.9 (8.4–13.8)	0.870	<0.001
peak $\dot{V}$CO_2_ (mL/min)	669 (558–774)	2112 (1739–2943)	0.870	<0.001
peak $\dot{V}$E (L/min)	22.8 (19.7–24.8)	63.3 (50.9–91.6)	0.870	<0.001
peak respiratory rate (breaths/min)	29.5 (26.4–32.3)	41.9 (37.3–46.8)	0.858	<0.001
peak METs	3.2 (2.9–3.9)	8.5 (7.5–10.2)	0.870	<0.001
Step count (steps)	688 (628–766)	—	—	—

Note: Values are presented as the median (interquartile range) or mean ± standard deviation, as appropriate. Changes from baseline were calculated as post-exercise minus pre-exercise values. Between-test differences in changes from baseline were assessed using paired-samples *t*-tests when the differences were normally distributed and Wilcoxon signed-rank tests when the differences were not normally distributed. r3MSST = Revised 3-Minute Seated Stepping Test; SBP = systolic blood pressure; DBP = diastolic blood pressure; peak VO_2_ = peak oxygen uptake; HR = heart rate; $\dot{V}$E = minute ventilation; peak VCO_2_ = peak carbon dioxide output; METs = metabolic equivalents. Dyspnea and lower-limb fatigue were assessed using the Borg 6–20 rating of perceived exertion scale. Effect size was expressed as Cohen’s d for paired-samples *t*-tests and as r for Wilcoxon signed-rank tests. Effect size (r) was calculated as Z/√N, where N represents the number of paired observations.

**Table 4:** Multiple Regression Analysis for Predicting Peak $\dot{V}$O_2_ Measured by CPET.

Variable	B	SE	β	*p*-Value
Sex	862.196	61.852	0.759	<0.001
Age (years)	−104.075	23.647	−0.240	<0.001
BMI (kg/m^2^)	42.022	12.819	0.190	0.002
Pre-exercise HR (beats/min)	−10.156	1.918	−0.295	<0.001
r3MSST step count (steps)	0.402	0.185	0.120	0.035

Model statistics: R^2^ = 0.858, adjusted R^2^ = 0.844, *p* < 0.001. Note: B = unstandardized regression coefficient; SE = standard error; β = standardized regression coefficient. Peak $\dot{V}$O_2_ = peak oxygen uptake; CPET = cardiopulmonary exercise testing; BMI = body mass index; HR = heart rate; r3MSST = Revised 3-Minute Seated Stepping Test.

## Data Availability

The data presented in this study are available on request from the corresponding author due to privacy and ethical restrictions.

## Abbreviations

The following abbreviations are used in this manuscript:

3MSST	3-Minute Seated Stepping Test
6MWT	Six-Minute Walk Test
BMI	Body Mass Index
CI	Confidence Interval
CPET	Cardiopulmonary Exercise Testing
CRF	Cardiorespiratory Fitness
DBP	Diastolic Blood Pressure
HR	Heart Rate
ICC	Intraclass Correlation Coefficient
ISWT	Incremental Shuttle Walk Test
MDC_95_	Minimal Detectable Change at the 95% Confidence Level
METs	Metabolic Equivalents
RMSE	Root Mean Square Error
RPP	Rate-Pressure Product
r3MSST	Revised 3-Minute Seated Stepping Test
SBP	Systolic Blood Pressure
SD	Standard Deviation
SEM	Standard Error of Measurement
$\dot{V}$CO_2_	Carbon Dioxide Output
VE	Minute Ventilation
VIF	Variance Inflation Factor
$\dot{V}$O_2_	Oxygen Uptake
